# A Warm, Stratified, and Restricted Labrador Sea Across the Middle Eocene and Its Climatic Optimum

**DOI:** 10.1029/2020PA003932

**Published:** 2020-10-09

**Authors:** Margot J. Cramwinckel, Helen K. Coxall, Kasia K. Śliwińska, Marcel Polling, Dustin T. Harper, Peter K. Bijl, Henk Brinkhuis, James S. Eldrett, Alexander J. P. Houben, Francien Peterse, Stefan Schouten, Gert‐Jan Reichart, James C. Zachos, Appy Sluijs

**Affiliations:** ^1^ Department of Earth Sciences, Faculty of Geoscience Utrecht University Utrecht The Netherlands; ^2^ Now at School of Ocean and Earth Science, National Oceanography Centre Southampton University of Southampton Southampton UK; ^3^ Department of Geological Sciences Stockholm University Stockholm Sweden; ^4^ Geological Survey of Denmark and Greenland, GEUS Copenhagen Denmark; ^5^ Now at Naturalis Biodiversity Center Leiden The Netherlands; ^6^ Department of Earth and Planetary Sciences University of California Santa Cruz CA USA; ^7^ Now at Department of Geology The University of Kansas Lawrence KS USA; ^8^ NIOZ Royal Netherlands Institute for Sea Research, Department of Marine Microbiology and Biogeochemistry, and Utrecht University Den Burg The Netherlands; ^9^ Shell International Exploration and Production B. V. Rijswijk The Netherlands; ^10^ Applied Geosciences Team Netherlands Organisation for Applied Scientific Research (TNO) Utrecht The Netherlands

## Abstract

Several studies indicate that North Atlantic Deep Water (NADW) formation might have initiated during the globally warm Eocene (56–34 Ma). However, constraints on Eocene surface ocean conditions in source regions presently conducive to deep water formation are sparse. Here we test whether ocean conditions of the middle Eocene Labrador Sea might have allowed for deep water formation by applying (organic) geochemical and palynological techniques, on sediments from Ocean Drilling Program (ODP) Site 647. We reconstruct a long‐term sea surface temperature (SST) drop from ~30°C to ~27°C between 41.5 to 38.5 Ma, based on TEX_86_. Superimposed on this trend, we record ~2°C warming in SST associated with the Middle Eocene Climatic Optimum (MECO; ~40 Ma), which is the northernmost MECO record as yet, and another, likely regional, warming phase at ~41.1 Ma, associated with low‐latitude planktic foraminifera and dinoflagellate cyst incursions. Dinoflagellate cyst assemblages together with planktonic foraminiferal stable oxygen isotope ratios overall indicate low surface water salinities and strong stratification. Benthic foraminifer stable carbon and oxygen isotope ratios differ from global deep ocean values by 1–2‰ and 2–4‰, respectively, indicating geographic basin isolation. Our multiproxy reconstructions depict a consistent picture of relatively warm and fresh but also highly variable surface ocean conditions in the middle Eocene Labrador Sea. These conditions were unlikely conducive to deep water formation. This implies either NADW did not yet form during the middle Eocene or it formed in a different source region and subsequently bypassed the southern Labrador Sea.

## Key Points

- The middle Eocene Labrador Sea was characterised by warm, low‐salinity surface waters
- We find two phases of superimposed sea surface warming and subtropical plankton incursions, including the Middle Eocene Climatic Optimum
- Bottom water stable carbon and oxygen isotope ratios indicate isolated basin

## 1 Introduction

Density‐driven sinking of North Atlantic Deep Water (NADW) together with wind‐driven surface ocean circulation powers the northern limb of today's global ocean overturning (e.g., Broecker, 1991; Wüst & Defant, 1936). The dominance of deep water formation in the Atlantic rather than Pacific Ocean is mainly due to the higher salinity of the Atlantic Ocean (de Boer et al., 2008; Ferreira et al., 2018). Sinking of water masses in the modern North Atlantic is primarily driven by low sea surface temperature (SST) and high sea surface salinity (SSS) in the two main source regions: the Nordic Seas (Norwegian‐Greenland Sea and Iceland Sea) and Labrador Sea (Dickson & Brown, 1994). Formation of NADW is a driving component of ocean circulation today, and the associated North Atlantic Current is responsible for significant northward transport of heat. However, the onset and strengthening of NADW formation remain poorly constrained. This is a crucial knowledge gap for paleovalidation of climate models that are ultimately used to predict future climate change.

**Figure 1:**
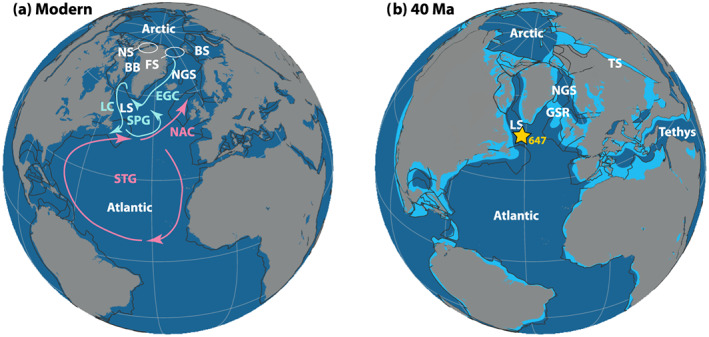
Modern and middle Eocene North Atlantic (paleo)geographic setting. (a) Modern‐day geography. Simplified surface ocean currents marked in light blue (cold) and pink (warm): EGC = East Greenland Current; LC = Labrador Current; NAC = North Atlantic Current; SPG = subpolar gyre; STG = subtropical gyre. Ocean basins and seaways marked in white: BB = Baffin Bay; BS = Barents Sea; FS = Fram Strait; LS = Labrador Sea; NGS = Norwegian‐Greenland Sea; NS = Nares Strait. Dark blue fill represents ocean crust, and black lines represent outlines of continental plates. Gray fills indicate modern coastlines. (b) Approximate paleogeographic reconstruction for 40 Ma, together with the paleolocation of ODP Site 647. Map produced with GPlates, using continental polygons and coastlines from Matthews et al. (2016) and the paleomagnetism‐based rotation frame of Torsvik et al. (2012). Selected oceanographic features from panel (a) annotated. Additional features in panel (b): TS = Turgay Strait; GSR = Greenland‐Scotland Ridge. Dark blue fill represents ocean crust, and black lines represent outlines of continental plates. Gray fills indicate coastlines, rotated together with the continental plates to a 40 Ma position. Light blue represents interpreted flooded continental shelf in the middle Eocene.

In the modern ocean, the densest components of NADW are formed in winter in the Norwegian‐Greenland Sea and enter the North Atlantic by flowing over the Greenland‐Scotland Ridge (GSR) (Quadfasel & Käse, 2007). These water masses are overlain by the less dense intermediate deep waters formed in the Labrador Sea in winter, formed in roughly equal proportion to the Nordic water mass (Dickson & Brown, 1994). Surface waters of the Labrador Sea form part of the Atlantic subpolar gyre (Figure 1a), which is a counterclockwise gyre consisting of the warm northward flowing North Atlantic Current at the eastern end and the cold southward flowing Baffin Current‐Labrador Current and the East Greenland Current at the western end (e.g., Rossby, 1999). The Labrador Sea has a fairly restricted connection to the Arctic Ocean through the Baffin Bay and Nares Strait, whereas the Norwegian‐Greenland Sea has broader surface water connections to the Arctic through the Fram Strait and Barents Sea (west and east of Svalbard, respectively) (Figure 1a) (Aagaard & Carmack, 1989).

A range of simple conceptual (Stommel, 1961) to complex IPCC‐class (Drijfhout et al., 2015) model simulations indicates high sensitivity of the global thermohaline circulation to surface ocean conditions in the North Atlantic Ocean, with multiple stable steady states. Formation of NADW has therefore been recognized as an important “tipping element” of Earth's climate system (Drijfhout et al., 2015; Lenton et al., 2008). Reorganizations of global ocean circulation caused by NADW formation shutdown due to fresh meltwater input played an important role in transient climate events during the last glacial period (McManus et al., 2004; Rahmstorf, 2002). As anthropogenic addition of carbon dioxide (CO_2_) to the atmosphere warms the planet, NADW formation might similarly diminish or shut down, with important consequences for global ocean circulation and ocean heat transport to northwestern Europe (IPCC, 2013, 2019). Some observational evidence indicates the Atlantic meridional overturning circulation (AMOC) is currently slowing down (Rahmstorf et al., 2015; Srokosz & Bryden, 2015), as the North Atlantic is freshening (Curry et al., 2003), which might be related to anthropogenic‐induced greenhouse forcing.

Although a crucial part of today's ocean circulation, the timing of the initiation of NADW, or of its precursor termed Northern Component Water (NCW), remains poorly constrained. Geological reconstructions of NCW formation derive from deep ocean contourite drift deposits as well as geochemical tracers of ocean circulation recorded in sediments and together indicate large uncertainty in timing of NCW onset and evolution. Contourite drift deposition offshore Newfoundland started around 47 Ma, indicating deep flow at the western bound of the North Atlantic, possibly by a weak north‐to‐south flowing Deep Western Boundary Current (Boyle et al., 2017). These contourites strengthened around 25 Ma to laminated mudwaves, suggestive of Deep Western Boundary Current intensification (Boyle et al., 2017). Drift deposits in the Faroe‐Shetland Basin have been interpreted as evidence of deep water overflow across the GSR from the earliest Oligocene (~35 Ma) (Southeast Faroes Drift, Davies et al., 2001; Wold, 1994) or possibly even the early Eocene (~50–49 Ma) (Judd Falls Drift, Hohbein et al., 2012). However, the nature of this water transport over the GSR, the depth history of the sill, and the degree of deep water recirculation between east and western North Atlantic basins at this time remains highly uncertain (Uenzelmann‐Neben & Gruetzner, 2018). Moreover, older interpretations attributed early Cenozoic North Atlantic drift deposits to southern‐origin deep waters (without GSR overflows) (e.g., Stoker, 1998), which likely dominated the Atlantic during the early Paleogene and still have a significant presence in the northeastern Atlantic today (Ferreira & Kerr, 2017; Johnson, 2008; Stoker, 1998).

Comparison of geochemical records from the North and South Atlantic indicates that southward export of NCW did not occur until the latest Eocene (~36–35 Ma) (Coxall et al., 2018) or earliest Oligocene (~33 Ma) (Via & Thomas, 2006). Geochemical records from the Bay of Biscay seem to indicate a transient switch to North Atlantic deep water formation during an early Eocene warming event (D'haenens et al., 2014), although this inference is based on the assumption that Eocene Atlantic north‐south carbon isotope (δ^13^C) gradients were similar to the modern, which may not have been the case (Coxall et al., 2018). Crucially, insufficient constraints on surface ocean conditions in potential NCW source regions hamper reconstructions of Eocene North Atlantic overturning regimes and thus our understanding of the global ocean circulation state in the warm Eocene. Importantly, Eocene boundary conditions were very different from today, with higher temperatures, intensified hydrological cycling, and a different shape and bathymetry of the Atlantic Ocean (e.g., Seton et al., 2012). Estimates of global mean temperature indicate this dropped from about 27–29°C in the early Eocene to 23–26°C during the middle Eocene and ~19°C during the late Eocene (Cramwinckel et al., 2018; Inglis et al., 2020). A warmer climate by itself results in enhanced hydrological cycling, with an increased contrast between regions of excess evaporation in the subtropics and excess precipitation at high latitudes (Held & Soden, 2006; Pierrehumbert, 2002). The Atlantic Ocean opened and progressively widened during the Cretaceous to Paleocene (Pitman & Talwani, 1972; Pérez‐Díaz & Eagles, 2017). During the Eocene, the basin was still much narrower than today (Figure 1b). Until magnetic Chron C13n (close to 34 Ma) seafloor spreading was occurring in the Labrador Sea and Nordic Seas (Chalmers & Pulvertaft, 2001; Roest & Srivastava, 1989). The depth of the GSR, as modulated by Icelandic mantle plume activity (Parnell‐Turner et al., 2014; Steinberger et al., 2019), controlled the connectivity between the Nordic Seas and Atlantic Ocean (Stärz et al., 2017).

Increased high‐latitude precipitation together with a relatively narrow and restricted northern North Atlantic basin geometry likely made regional surface waters relatively fresh (Hutchinson et al., 2018; Roberts et al., 2009). Indeed, reconstructions indicate low SSS in the early‐middle Eocene Arctic Ocean that periodically sustained blooms of the floating freshwater fern *Azolla* (Brinkhuis et al., 2006; Waddell & Moore, 2008). The occurrence of age‐equivalent *Azolla* in sediment cores through the Nordic Seas and northeastern North Atlantic shows that Arctic surface waters were at least periodically exported (Barke et al., 2012; Collinson et al., 2013; Eldrett et al., 2004).

A suite of model simulations supports a high sensitivity of NCW formation to both climatic (Roberts et al., 2011; Speelman et al., 2010) and tectonic (Cope & Winguth, 2011; Elsworth et al., 2017; Hutchinson et al., 2018; Roberts et al., 2009; Vahlenkamp et al., 2018) boundary conditions, through effects on surface ocean parameters in the North Atlantic and Nordic Seas. With open pan‐Arctic gateways, high freshwater fluxes into the North Atlantic would cause salinity stratification (Baatsen et al., 2018; Hutchinson et al., 2018, 2019), preventing strong AMOC‐type overturning.

Here we assess water column conditions and thereby overturning state in one of the possible source regions for NCW, the Labrador Sea (Figure 1). We use sediments from Ocean Drilling Program (ODP) Site 647 in the southern Labrador Sea with the aim of reconstructing surface and deep ocean temperature and salinity (Figure 1). Geochemical reconstructions at this site indicate the presence of poorly ventilated, high‐nutrient bottom waters during the late Eocene, between 37.5 and 34.4 Ma (Coxall et al., 2018). We target sediments from a period prior to this, between 41.5 and 38.5 Ma. Global reconstructions of ocean temperature (Cramwinckel et al., 2018; Evans et al., 2018; Zachos et al., 2008) display this as a period of gradual cooling occurring halfway between the Early Eocene Climatic Optimum (EECO; ~52–50 Ma) and the Eocene‐Oligocene Transition (EOT; ~34 Ma). Superimposed on global cooling, the time interval studied here also includes the Middle Eocene Climatic Optimum (MECO) at ~40 Ma, a half‐million‐year episode of enhanced warmth (Bohaty et al., 2009; Bohaty & Zachos, 2003). The MECO was likely driven by a volcanic‐induced imbalance in the long‐term carbon cycle (Bijl et al., 2010; Sluijs et al., 2013; van der Ploeg et al., 2018) causing modest CO_2_ rise (Henehan et al., 2020) and as such is expected to have a global signature. We employ the MECO to assess sensitivity of North Atlantic surface parameters to different climatic boundary conditions. Climatic and environmental change during the MECO has been reconstructed at a wide range of locations including the South Atlantic, Indian, Tethys, and Pacific Oceans (e.g., Bohaty et al., 2009; Boscolo‐Galazzo et al., 2014; Giorgioni et al., 2019; Henehan et al., 2020; Villa et al., 2014), but constraints from the northern North Atlantic ocean are lacking. Eocene sediments at Site 647 contain both well‐preserved, “glassy”, carbonate microfossils (Arthur, Srivastava, et al., 1989; Pearson & Burgess, 2008) and abundant, well‐preserved organic microfossils (Firth et al., 2012; Head & Norris, 1989). Therefore, this site is highly suitable for multiproxy reconstruction of marine conditions. Here, we produce reconstructions of surface ocean and seafloor conditions in the middle Eocene Labrador Sea, in order to yield critical information on northwest Atlantic circulation regimes, both during the middle Eocene and in response to superimposed warming. We reconstruct SST and SSS based on organic (TEX_86_) and inorganic (δ^18^O and Mg/Ca) geochemical proxies in conjunction with analysis of microfossil assemblages, specifically planktonic foraminifera and organic dinoflagellate cysts (dinocysts).

## 2 Materials and Methods

### 2.1 Material

ODP Site 647 (53°19.876′N, 45°15.717′W, middle Eocene latitude was ~50°N; paleolatitude.org Version 2.1; Van Hinsbergen et al., 2015, using the paleomagnetic reference frame of Torsvik et al., 2012, and the geological reconstruction of Seton et al., 2012) is located in the southern Labrador Sea (Figure 1). The site was drilled on the southern flank of the (late Neogene) Gloria Drift, at a present‐day water depth of 3,862 m. The site was likely located at similar bathyal water depths of ~2000–3,000 m in the middle Eocene (Srivastava et al., 1987; updated analysis in Coxall et al., 2018). Basaltic basement at 700 m below seafloor (mbsf) underlies a succession of early Eocene to Holocene sediments (Srivastava et al., 1987). The sediments studied here roughly span the interval 400–500 mbsf and are composed of nannofossil claystones that were deposited under an average sedimentation rate of 3.6 cm/kyr (Arthur, Srivastava, et al., 1989; Srivastava et al., 1987).

The lithology of the sediments consists mostly of clay, containing calcium carbonate (35 ± 12 wt. %), some TOC (0.2 ± 0.05 wt. %), and common mineral concretions, including glauconite, pyrite, and authigenic carbonates (Arthur, Srivastava, et al., 1989; Srivastava et al., 1987). While the overlying lower Oligocene sediments also contain abundant biogenic silica, this has been converted to opal‐CT in middle‐upper Eocene sediments (Arthur, Srivastava, et al., 1989). Clay mineralogy is dominated by smectite (>70% of clay) with some illite and kaolinite, and geochemistry of the sediments is similar to modern North Atlantic abyssal red clays. The source of clay is interpreted to be predominantly terrigenous (Arthur, Srivastava, et al., 1989; Nielsen et al., 1989; Srivastava et al., 1987). These sediments are described as hemipelagic, lacking the turbiditic deposits that have been found in younger sediments at the same site, and with no other indicators for strong off‐shelf transport (Srivastava et al., 1987). Drift deposition at the site, forming the Gloria Drift, initiated much later in the late Neogene, mainly in the upper Pliocene and Pleistocene (Srivastava et al., 1987; Uenzelmann‐Neben & Gruetzner, 2018).

For age control, we follow the integrated biomagnetostratigraphic age model of Firth et al. (2012). Based on this age model, the presence of a seemingly complete representation of the MECO was identified around 450–460 mbsf in Hole 647A (Firth et al., 2012). An age‐depth plot is presented in Firth et al. (2012) and indicates no large changes in sedimentation rate over this interval. We do note that the middle Eocene magnetostratigraphy is not fully constrained, and some biostratigraphic data are inconclusive and might have been affected by unusual environmental conditions. Importantly however, Chron C19n and C18n.1n do seem reliably resolved, and sediments in between should cover the MECO interval. We therefore present our results in the depth domain, with the available age constraints plotted as a secondary axis.

### 2.2 Palynology

A total of 37 samples from ODP Hole 647A was processed for palynology. A known amount of a *Lycopodium clavatum* spore standard was added to crushed, oven‐dried (60°C), and weighted (10–20 g dry weight) sediment samples, in order to be able to quantify dinocyst content in terms of absolute number of dinocyst counts per gram (c.p.g.) sediment. Samples were treated with 30% HCl and ~38–40% HF to dissolve carbonates and silicates, respectively. After each step, samples were washed with water, settled, and decanted. The remaining residue was sieved over nylon mesh sieves of 250 and 10 μm. The resulting 10–250 μm fraction was subjected to an ultrasonic bath to break up agglutinated particles. A drop of homogenized residue was mounted on a glass microscope slide with glycerin jelly and sealed. All slides are stored in the collection of the Laboratory of Palaeobotany and Palynology, Department or Earth Sciences, Utrecht University. Palynomorphs were counted up to a minimum of 200 identified dinocysts. Dinocyst taxonomy as cited in Williams et al. (2017) was generally followed, with the exception of the Wetzellioid family, for which the suggestions made in Bijl et al. (2016) were followed (i.e., using the taxonomy of Fensome & Williams, 2004). Dinocyst paleoecological interpretations follow Brinkhuis (1994), Pross and Brinkhuis (2005), Sluijs and Brinkhuis (2009), and Frieling and Sluijs (2018). Grouped palynomorph abundances are reported as percentages of total palynomorphs, ±1 sd. Dinocyst abundances are likewise reported as percentages of total dinocysts, ±1 sd.

### 2.3 Organic Geochemistry

A total of 59 samples from ODP Hole 647A was processed for TetraEther indeX of tetraethers consisting of 86 carbon atoms (TEX_86_) palaeothermometry. Organic compounds were extracted from freeze‐dried, powdered samples (~10–14 g dry weight) with dichloromethane (DCM):methanol (MeOH) (9:1, v:v) using a Dionex accelerated solvent extractor. Lipid extracts were subsequently separated by Al_2_O_3_ column chromatography into apolar, ketone, and polar fractions, using hexane:DCM (9:1, v/v), hexane:DCM (1:1, v/v), and DCM:MeOH (1:1, v/v), respectively. The polar fraction, including glycerol dialkyl glycerol tetraethers (GDGTs), was subsequently dissolved in hexane:isopropanol (99:1, v/v) and filtered using a 0.45 μm polytetrafluoroethylene (PTFE) filter. Lipid extraction and column chromatography occurred in three distinct batches: one batch at the Netherlands Institute for Sea Research in 2012 and two batches at Utrecht University in 2011 and 2018 (supporting information Data Set S1). The filtered polar fractions of the three batches were analyzed as one set using ultrahigh‐performance liquid chromatography/mass spectrometry (UHPLC/MS) following Hopmans et al. (2016), at Utrecht University, in order to quantify abundance of isoprenoid GDGTs (isoGDGTs) and branched GDGTs (brGDGTs). Samples with very low concentrations (i.e., peak area <3,000 mV/s and/or peak height <3× background signal) of any GDGT included in TEX_86_ were excluded from analysis. Based on relative abundances of GDGTs, the TEX_86_ and Branched versus Isoprenoid Tetraether (BIT) index values were calculated following Schouten et al. (2002) and Hopmans et al. (2004), respectively. The degree of cyclisation and the IR of brGDGTs were calculated to determine the sources of brGDGTs (De Jonge et al., 2014; Sinninghe Damsté, 2016), after which the BIT index was used to assess the contribution of terrestrially derived isoGDGTs that might disturb the TEX_86_‐SST relationship. Furthermore, several isoGDGT ratios were calculated to evaluate isoGDGT sourcing. These include the methane index (MI) (Zhang et al., 2011), GDGT‐2/crenarchaeol (Weijers et al., 2011), GDGT‐0/crenarchaeol (Blaga et al., 2009), and GDGT‐2/GDGT‐3 (Taylor et al., 2013) ratios, chosen to investigate potential contributions by methanotrophic, methanogenic, and deep‐dwelling GDGT producers to the measured GDGT pool, respectively. Analytical precision for TEX_86_ is ±0.3°C (±1 sd), based on long‐term observation of an in‐house standard at Utrecht University.

Several calibrations exist to translate TEX_86_ to SST, based on core‐top data sets and mesocosm experiments. Core‐top‐based calibrations have the advantage of implicitly integrating ecological complexity and other real‐world noise. Since part of our data is above the range of TEX_86_ values included in the modern core‐top data set (Kim et al., 2008; Tierney & Tingley, 2015)—which ranges to about 0.72 when excluding the anomalous data from the Red Sea (Trommer et al., 2009)—the choice between linear and exponential calibration models is relevant (see discussion in Cramwinckel et al., 2018; Hollis et al., 2019). Following the recommendations of Hollis et al. (2019), we present fractional GDGT abundances in the Data Set S1, to facilitate recalculation of SST from our data using different calibrations. In this study, we estimate SST using an exponential calibration ( ${\mathrm{TEX}}_{86}^{H}\;$ of Kim et al., 2010; calibration uncertainty ±2.5°C) and a linear calibration (O'Brien et al., 2017; calibration uncertainty ±2.0°C), both based on core‐top data.

### 2.4 Foraminifera: General Assemblage Characteristics, Species Selection, and Preservation

**Figure 2:**
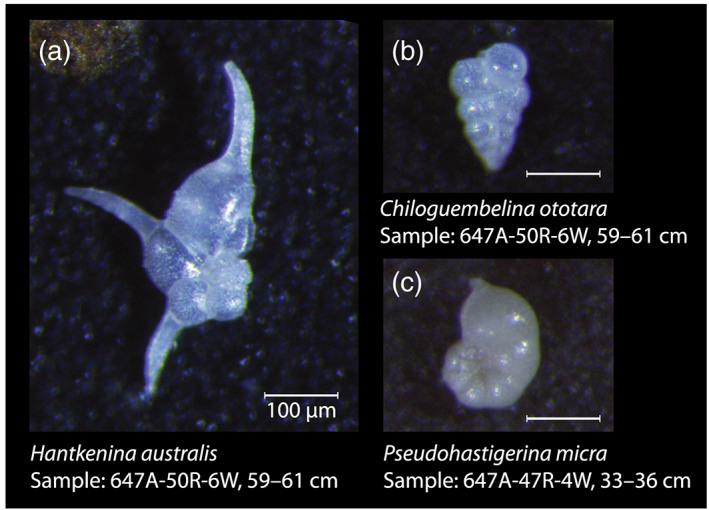
Light microscope images of representative Site 647 planktic foraminifera analyzed in this study. (a) *Hantkenina australis* (Sample 647A‐50R‐6, 59–61 cm), (b) *Chiloguembelina ototara* (Sample 647A‐50R‐6, 59–61 cm), and (c) *Pseudohastigerina micra* (Sample 647A‐47R‐4, 33–36 cm). Images were taken at Stockholm University with a Leica M205C binocular light microscope equipped with a Leica camera system. Scale bars all 100 μm. Note the small size of *P. micra* and *C. ototara* relative to *H. australis*. The shiny transparent appearance of the test calcite, revealing original fine surface details, signals excellent shell calcite preservation.

**Figure 3:**
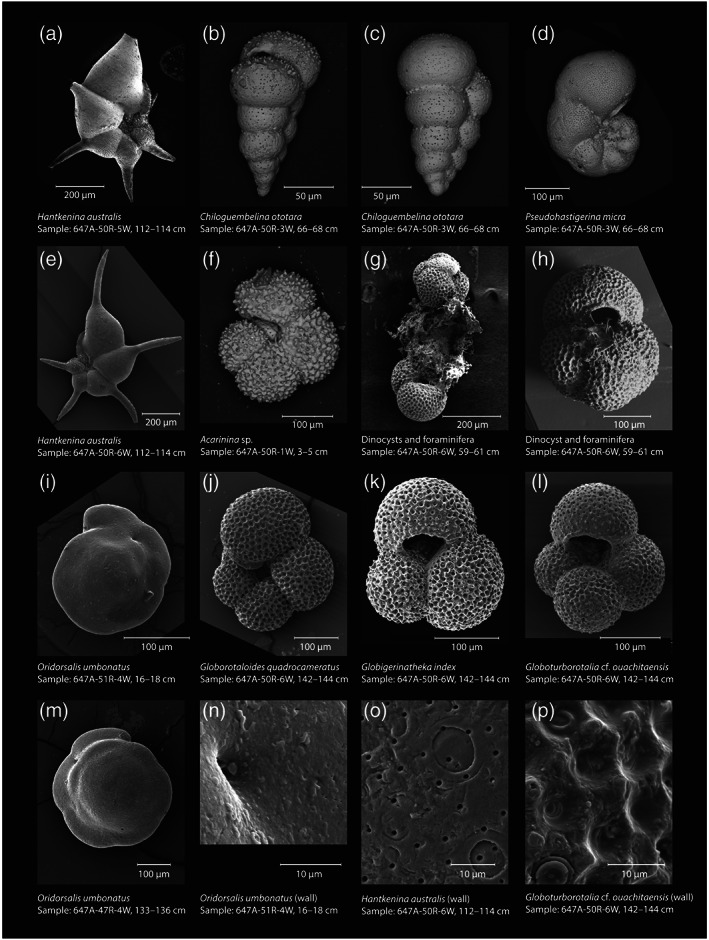
Scanning electron microscope (SEM) images of representative Site 647 microfossils. Taxon and sample names annotated in figure. Images (a)–(d) and (f)–(h) were taken with a Thermo Scientific Apreo SEM (uncoated, 10 kV, working distance 10 mm) at the University of California, Santa Cruz. Images (e), (j)–(l), (o), and (p) were taken with a Philips XL30 FEG ESEM (gold coated, 10 kV, spot size −3, working distance 9 mm) at Stockholm University. Images (i), (m), and (n) were taken with a Philips XL30 FEG ESEM (gold coated) at the School of Earth and Ocean Science at Cardiff University.

Middle Eocene foraminifera from Site 647 are, except for the MECO interval, excellently preserved (e.g., Pearson & Burgess, 2008) (Figures 2 and 3), likely related to the high clay content of the sediments. The primary planktonic signal carriers picked for stable isotope and trace element analyses are *Chiloguembelina ototara* and *Pseudohastigerina micra*, which were the most consistently present surface dwellers (Pearson et al., 2006) in our samples. These are relatively small‐sized taxa and were picked from the 63–150 μm fraction (Figure 2). Supplementary planktonic species were picked where available and include several acarininids, *Globigerinatheka index*, *Turborotalia pomeroli*, and *Hantkenina australis*. The primary benthic signal carrier is (the shallow infaunal) *Oridorsalis umbonatu*s, since *Cibicidoides* and *Nuttallides* spp. are rare. Samples were sieved in deionized water over a 63 μm mesh sieve. A low‐resolution planktonic foraminifera taxonomic study was performed on 12 samples from Cores 41R–53R (~390–500 mbsf) to broadly characterize assemblages. Taxonomy followed Pearson et al. (2006). Both the 63–150 and >150 μm fractions were assessed. Species abundance was qualitatively recorded in a range chart, as rare (R), few (F), common (C), or abundant (A). Additional qualitative estimates of foraminiferal preservation state (M = moderate, G = good, and Ex = excellent), the abundance of total foraminifera and foraminiferal fragments, and % benthic foraminifera were also recorded (Data Set S1).

For chemical analyses, foraminifera were picked from a higher‐resolution sample set of 29 samples. Samples with larger foraminifera (>150 μm) were lightly crushed; samples with smaller foraminifera (<150 μm) were not (Data Set S1). Samples that contained sufficient specimens were split into two fractions: one for stable isotope analysis and a second for trace element analysis. Samples with low numbers of individuals were measured for stable isotopes only.

### 2.5 Foraminifera: Stable Carbon (δ^13^C) and Oxygen (δ^18^O) Isotope Analysis

For stable isotope analysis, planktonic foraminifera (at least 20 μg) were analyzed at the University of California, Santa Cruz (USA), on a Thermo MAT 253 IRMS coupled to a Kiel IV carbonate device. Foraminifera were cleaned without using oxidative or reductive steps. Based on long‐term replicate measurements of consistency standards, analytical precision is ±0.05‰ for δ^13^C and ±0.08‰ for δ^18^O (±1 sd). Some of the smallest samples encountered pressure balancing issues, which caused an estimated additional ±0.1‰ uncertainty. Benthic foraminifera were measured using a Europa Geo 20‐20 mass spectrometer equipped with an automatic carbonate preparation system (CAPS) at the National Oceanography Centre, Southampton, UK. Analytical precision for these is ±0.03‰ for δ^13^C and ±0.07‰ for δ^18^O (±1 sd). Correction factors following Katz et al. (2003) were applied in order to convert *O. umbonatu*s isotope values to *Cibicidoides* equivalents.

### 2.6 Foraminifera: Trace Element Analyses (Whole Specimen Solution Based)

For determining foraminifera test trace element contents, we used 15–20 μg of material and applied the method developed specifically for small sample sizes by Rongstad et al. (2017). In short, this method uses an oxidative cleaning step and heat rinse, but omits a reductive cleaning step, which can incur considerable loss of valuable shell material. The cleaned planktonic foraminiferal samples were dissolved in 400 μl, 0.075 N HNO_3_ and measured for elemental composition at the University of California, Santa Cruz (USA), using ICP‐MS on a Thermo Element XR. The number of elements included in the analysis was limited due to small sample size. Contamination by adhering clays and/or carbonates was assessed using Fe/Ca and Mn/Ca ratios. Notably, while foraminiferal shells looked pristine under light and scanning electron microscopy (Figures 2 and 3), concentrations of contaminants Mn (2–4 mmol/mol) and Fe (0.6–1.6 mmol/mol) are quite high (Figure S1) compared to typical limit values of 0.1 mmol/mol for foraminiferal calcite.

## 3 Results

### 3.1 Palynology: Assemblages

**Figure 4:**
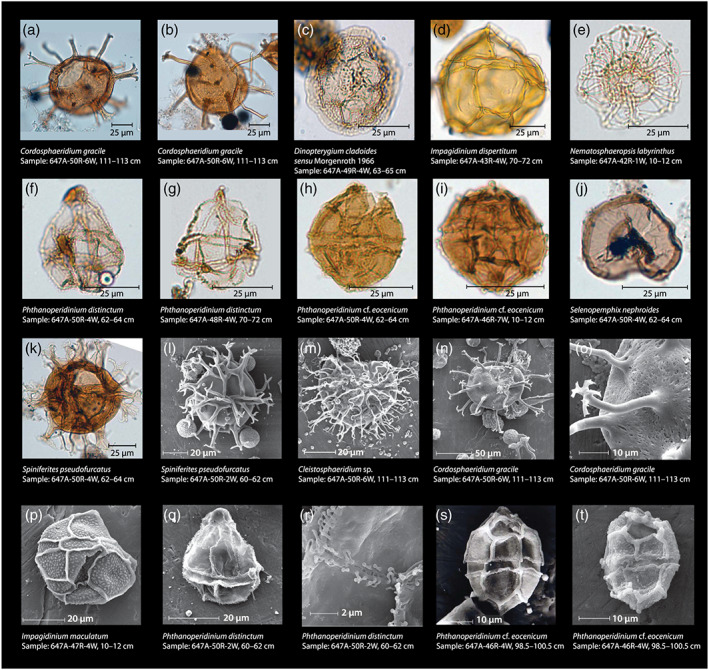
Light microscope (LM) and scanning electron microscope (SEM) images of representative Site 647 dinocysts. Taxon and sample names in figure. LM images (a–k) taken at Utrecht University with a light microscope equipped with a Leica camera system. SEM images (l–t) taken at Utrecht University with a Philips XL30 FEG ESEM (platinum coated).

**Figure 5:**
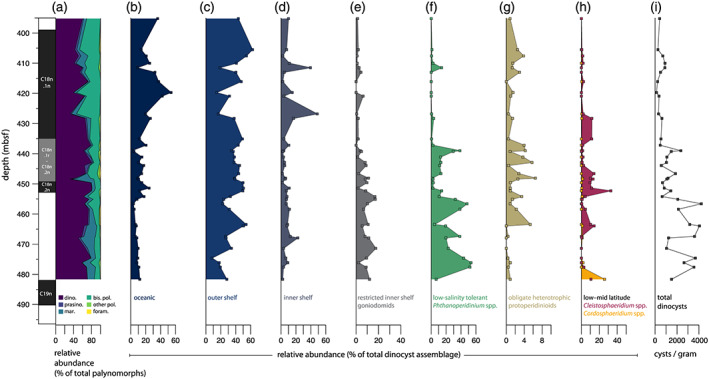
Palynomorph and dinocyst assemblages across the middle Eocene at Site 647. (a) Relative abundance of main encountered groups of palynomorphs, stacked area diagram plotted as percentage of total palynomorphs. dino. = dinocysts; prasino. = prasinophytes; mar. = other marine palynomorphs (other algal remains and prasinophytes); bis. pol. = bisaccate pollen; other pol. = other pollen and spores; foram. = organic benthic foraminiferal linings. (b–h) Relative abundance of representative groups plotted as percentage of total dinocysts. (i) Dinocyst content in cysts per gram of dry sediment. Plotted against depth in meters below seafloor.

The recovered palynological associations are dominated by well‐preserved, rich, and diverse dinocyst assemblages (average 63% ± sd 13% of total palynomorphs; Figures 4 and 5), with additional contributions by marine acritarchs (average 12 ± 10% of total palynomorphs), and bisaccate (gymnosperm) pollen (average 21 ± 11% of total palynomorphs). Other components, such as remains of green algae, angiosperm pollen, and spores, are only minor components of the palynological assemblage.

Dinocyst assemblages consist of a range of inner shelf to oceanic taxa (following Pross & Brinkhuis, 2005; Frieling & Sluijs, 2018). The generalist taxon *Spiniferites* complex (cpx.), which has higher relative abundances on the outer shelf (following Frieling & Sluijs, 2018), is fairly abundant (21 ± 9.5% of total dinocysts) (Data Set S1). Midshelf genera such as *Cerebrocysta* and *Corrudinium* (6.3 ± 5.0%), *Elytrocysta* and *Histiocysta* (5.8 ± 9.8%), *Cleistosphaeridium* (4.9 ± 6.8%) and *Enneadocysta* (2.7 ± 4.2%), and inner shelf epicystal Goniodomidae (6.2 ± 5.0%) are also present. The oceanic dinocysts *Impagidinium* spp. and *Nematosphaeropsis labyrinthus* (Dale & Dale, 1992) are consistently present, together forming 16 ± 12% of total dinocysts. Absolute dinocyst content ranges from ~130 to ~4,200 c.p.g. sediment. A shift can be observed around 455 mbsf, with an average of ~2,500 c.p.g. before to ~800 c.p.g. after this. Dinocysts derived from the obligate heterotrophic Protoperidinium family (Sluijs et al., 2005) are consistently present in low relative abundances (~5%) (Figure 5). Dinocysts of the genus *Phthanoperidinium*, interpreted to be adapted to lower than normal marine salinities (Barke et al., 2011; Frieling & Sluijs, 2018; Sluijs & Brinkhuis, 2009), are particularly abundant in the older part (>39.7 Ma) of the studied record. As a whole, the dinocyst assemblage is similar to existing middle Eocene dinocyst records from the Nordic Seas (Eldrett et al., 2004). Superimposed on the background assemblage are several acmes. The most prominent are a *Cleistosphaeridium* spp. incursion around 452 mbsf and a *Cordosphaeridium gracile* incursion around 482 mbsf (Figures 4 and 5). These taxa are typically considered to indicate a low‐latitude to midlatitude habitat that might be rooted in temperature preference (e.g., Bijl et al., 2011).

### 3.2 Organic Geochemistry: GDGT Distributions

**Figure 6:**
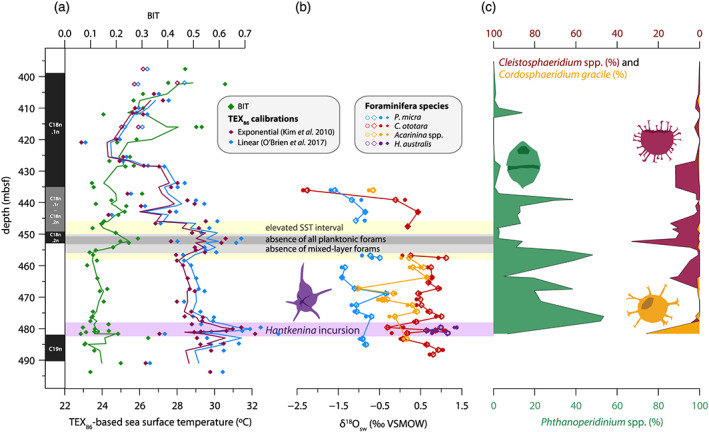
Middle Eocene surface ocean conditions at Site 647. (a) TEX_86_‐based sea surface temperature (°C) (exponential calibration of Kim et al., 2010, in red; linear calibration of O'Brien et al., 2017, in blue) and BIT index (green); 3‐point moving averages plotted as thick lines. Open symbols represent TEX_86_‐based SST values with BIT > 0.4. Propagated analytical plus calibration uncertainty is ±2.6°C for the Kim et al. calibration and ±2.1°C for the O'Brien et al. calibration. (b) Inferred δ^18^O of surface ocean (converted to ‰ VSMOW) calculated from ${\mathrm{TEX}}_{86}^{H}$‐based SST (open symbols: LOESS fit of ${\mathrm{TEX}}_{86}^{H}$‐based SST; closed symbols: linear interpolation of ${\mathrm{TEX}}_{86}^{H}$‐based SST) and planktic foraminiferal δ^18^O (Figure 7), using the Erez and Luz (1983) (circles) and Kim and O'Neil (1997) (diamonds) δ^18^O‐to‐temperature calibrations. Open diamonds are connected with a line. Note that using ${\mathrm{TEX}}_{86}^{H}$‐based SST likely overestimates the temperature effect on δ^18^O_foram_, and thus underestimates the salinity effect, as ${\mathrm{TEX}}_{86}^{H}$ is calibrated based on the modern relationship between GDGTs in core‐top sediments and satellite‐derived SSTs. The studied foraminifera would have lived at deeper, somewhat cooler depths than the depth represented by satellite‐derived SSTs. (c) Relative abundance (% of total dinocyst assemblage) of selected dinocyst species. Low‐salinity tolerant *Phthanoperidinium* spp. in green, relative abundance plotted from left to right. Low‐latitude/midlatitude *Cleistosphaeridium* spp. (red) and *Cordosphaeridium gracile* (purple) relative abundance plotted from right to left. Gray horizontal shading refers to the interval of absent mixed‐layer planktonic foraminifera, with dark gray representing total absence of planktic species. Purple horizontal shading denotes the interval of *H. australis* incursion. Plotted against depth in meters below seafloor.

The measured GDGTs consist of 76 ± 10% isoGDGTs and 24 ± 10% brGDGTs. The isoGDGT distributions indicate that these components were primarily produced by surface ocean‐dwelling Thaumarchaeota, without elevated concentrations of specific isoGDGTs pointing to enhanced GDGT contributions by methanotrophic or methanogenic microbes (Blaga et al., 2009; Weijers et al., 2011; Zhang et al., 2011), deep ocean‐dwelling archaea (Taylor et al., 2013), or modern Red Sea‐like archaeal populations (Inglis et al., 2015; Trommer et al., 2009) (Data Set S1). brGDGT‐Ia is present in high abundance relative to brGDGT‐Ib and Ic (Data Set S1), suggesting a soil source of brGDGTs (Sinninghe Damsté, 2016). BIT index values can therefore be interpreted as a measure for the abundance of river‐transported continental‐derived GDGTs relative to marine GDGTs (Hopmans et al., 2004; Zell et al., 2013). The BIT index is higher than 0.4 for only five out of our 59 samples (Figure 6 and Data Set S1). Although this indicates a predominantly marine source of isoGDGTs for most of the samples, there is a significant (*p* < 0.0001) correlation between TEX_86_ and BIT values (Figure S2). Because the correlation exists at BIT index values <0.3 and even <0.2, this might indicate that this correlation reflects a true environmental connection between terrestrial biomarker contributions and climate rather than merely a terrestrial overprint of the isoGDGT pool. To be conservative, we nevertheless excluded TEX_86_ data points for which BIT is above a threshold value of 0.4 (following Weijers et al., 2006; five samples in total) for SST analysis. Unfortunately, brGDGT contents were unsuitable for brGDGT‐based paleothermometry (De Jonge et al., 2014; Weijers et al., 2007), with cyclopentane moiety‐containing brGDGTs below detection limit in all samples. Fractional abundances of 6‐methyl brGDGTs isomers relative to the sum of 5‐methyl and 6‐methyl brGDGTs, as described in the isomer ratio (IR) (De Jonge, Stadnitskaia, et al., 2014; Sinninghe Damsté, 2016), are relatively high (Data Set S1). Especially the relative abundance of IIIa′ relative to IIIa is high, resulting in IR_hexa_ values of 0.62 ± 0.09. Values of IR_penta_ are much lower, 0.34 ± 0.05, and do not correlate to IR_hexa_ (Data Set S1).

### 3.3 Organic Geochemistry: Trends and Patterns in GDGTs

Calculated SSTs based on the nonlinear ${\mathrm{TEX}}_{86}^{H}$ calibration (Kim et al., 2010) and linear calibration of O'Brien et al. (2017) cover a range of temperatures from 24°C to 31°C (Figure 6a). Two warming phases are apparent in our record, both with a magnitude of about 2°C. The older of these occurs around the C19n‐C18r boundary, corresponding to ~41.1 Ma, and reaches peak SSTs of 31–32°C. The younger phase of warming occurred around 458–452 mbsf, reaching peak SSTs of 30–31°C. The timing of this younger warming, near the well‐resolved C18r‐C18n.2n boundary (Firth et al., 2012) close to 40.1 Ma, is consistent with the MECO. The MECO is followed by a phase of strong cooling, with minimum temperatures of 23–24°C around 420–425 mbsf. Finally, the youngest part of the record suggests warming between ~420 and ~405 mbsf. Although BIT indices are generally below 0.3 in the studied interval, BIT values increase slightly during the MECO, and there is an increasing trend in the younger, post‐MECO part of the record (Figure 6a). IR_hexa_ shows a somewhat increasing trend throughout the record, whereas IR_penta_ has a stable background value with superimposed peaks (Data Set S1).

### 3.4 Planktonic Foraminifera: Preservation and Assemblages

**Figure 7:**
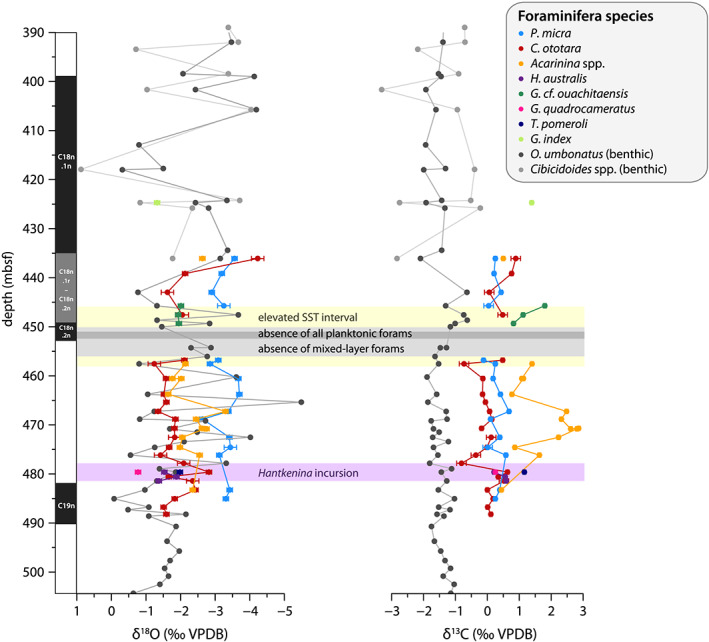
Foraminiferal δ^18^O and δ^13^C at Site 647. Planktic and benthic δ^18^O (left) and δ^13^C (right) (‰ VPDB) for several species. Error bars denote ±1 sd. Error is slightly higher for some samples that had very small mass. Full names of planktic foraminifera are as follows: *Pseudohastigerina micra*, *Chiloguembelina ototara*, *Acarinina* spp., *Hantkenina australis*, *Globoturborotalita* cf. *ouachitaensis*, *Globorotaloides quadrocameratus*, *Turborotalia pomeroli*, and *Globigerinatheka index*. These are mixed‐layer dwellers, except for *T. pomeroli* and *G. quadrocameratus*, which were likely thermocline and subthermocline dwellers, respectively. Full name of benthic foraminifera is *Oridorsalis umbonatus*. Colors as in legend. Gray horizontal shading refers to the interval of absent mixed‐layer planktonic foraminifera, with dark gray representing total absence of planktic species. Purple horizontal shading denotes the interval of the *H. australis* incursion. Plotted against depth in meters below seafloor.

The middle Eocene interval at Site 647 between ~390 and 500 mbsf contains well‐preserved to exceptionally well‐preserved (glassy) planktonic foraminifera, except for an interval of severe dissolution around the MECO interval (~450–456 mbsf), as revealed by the higher‐resolution isotopic sample set, where planktonic foraminifera are almost absent (Data Set S1 and Figure 7). These same samples are characterized by lack of surface‐dwelling planktonic species such as *Acarinina* spp., *P. micra*, and *C. ototara*, leaving only deeper‐dwelling planktonic and benthic taxa. The two samples at 452.45 and 451.03 mbsf (dated at 40.13 and 40.09 Ma) are completely devoid of planktonic foraminifera, leaving only benthic foraminifera. These samples correspond to the interval of peak TEX_86_ values. In contrast, foraminifera show no signs of dissolution during the older peak in TEX_86_ values around 41.1 Ma. Assemblages are of relatively low diversity compared to lower‐latitude regions, and average test sizes are noticeably smaller, with large specimens/species (>300 μm) being uncommon. The most conspicuous species, in abundance order (high to low), are *T. pomeroli*, *Turborotalia frontosa*, *Catapsydrax unicavus*, *Globorotaloides eovariabilis*, *Globorotaloides quadrocameratus*, *Subbotina eocaena*, *Globoturborotalita bassriverensis*, and *Globoturborotalita ouachitaensis* (Table S1). *Acarinina collactea* and *Acarinina medizzea*, *P. micra*, *C. ototara*, and *Paragloborotalia nana* persist at relatively low levels, mostly in the 63–150 μm size fraction. Also notable, and occurring in the smaller size fraction, are two species of *Turborotalita*, that is, *Turborotalita praequinqueloba* and *Turborotalita carcoselleensis*. The tropical genus *Hantkenina* is scarce, apart from a short interval in Core 50R where the distinctive species *H. australis* occurs, becoming common in Sample 647A‐50R‐6, 59–61 cm. Species belonging to *Morozovelloides* and the *Turborotalia cerroazulensis* group, common at lower latitudes, are almost entirely absent. Globigerinathekids, abundant at southern high latitudes during this interval (Huber, 1991), are also relatively uncommon, although *G. index* makes an appearance (sufficient for a stable isotope sample) from 450 mbsf above the MECO horizon. Assemblages are unusual in the persistence of *Globorotaloides*, both *G. quadrocameratus* and *G. eovariabilis*, the former having been originally described from this location (Olsson et al., 2006). The 41.1 Ma warming interval coincides with an incursion of several (well‐preserved) low‐latitude taxa including more diverse acarininids and *Hantkenina*, as observed previously by Srivastava et al. (1987). These hantkeninids are now assigned to the species *H. australis* Finlay, based on the slightly backward curving tubulospines (Figures 2a, 3a, and 3e) (Pearson & Burgess, 2008, after Coxall & Pearson, 2006). These examples are remarkably pristine looking, both in terms of the high‐quality of the test surface preservation and the intact nature of the protruding tubulospines. Other observations in the planktonic foraminifera residues include concentrations of authigenic minerals, including pyrite throughout and rusty brown mineral aggregates (e.g., Sample 45R‐1, 60–62 cm [424.70 mbsf]), likely authigenic iron, and/or manganese carbonates, implying reducing conditions at the seafloor or within the upper few centimeters of seafloor sediments (Arthur, Dean, et al., 1989). Our coarse estimates of the benthic: planktonic ratio suggest that benthic foraminifera make up ~2–5% of the total foraminifera assemblage up to 470.70 mbsf and thereafter increase to 10–50%. Of these, agglutinated species make up around 80% in Cores 55R and 46R (Kaminski et al., 1989).

### 3.5 Planktonic Foraminifera: Stable Isotope Chemistry

All analyzed planktonic foraminifera species are characterized by low δ^18^O values of <0‰ VPDB (Figure 7). Values for *P. micra* are most depleted in ^18^O (between −4‰ and −3‰ VPDB), while *C. ototara* and *H. australis* values are mostly between −3‰ and −1‰ VPDB. δ^18^O values for *Acarinina* spp. are intermediate between *P. micra* and *C. ototara*. In terms of δ^13^C, *C. ototara*, *P. micra*, and *H. australis* record similar values of about −1‰ to 1‰, with values for *C. ototara* overall a bit lower than *P. micra* (Figure 7). *Acarinina* spp. δ^13^C values are more enriched in ^13^C and exhibit a large range of values between 0.5‰ and 3‰. The different species show large variability but no clear common trends or peaks in δ^18^O or δ^13^C over the studied interval, suggesting dynamic environmental conditions on a timescale shorter than that of the sampling resolution, which is 70 kyr on average. The strong negative δ^18^O and positive δ^13^C peaks in *Acarinina* spp. in the interval 473–465 mbsf are enigmatic, as they seem unrelated to different morphospecies or different size fractions of *Acarinina* (Data Set S1). Values for the MECO interval are lacking, as the studied species were not present in those samples.

**Figure 8:**
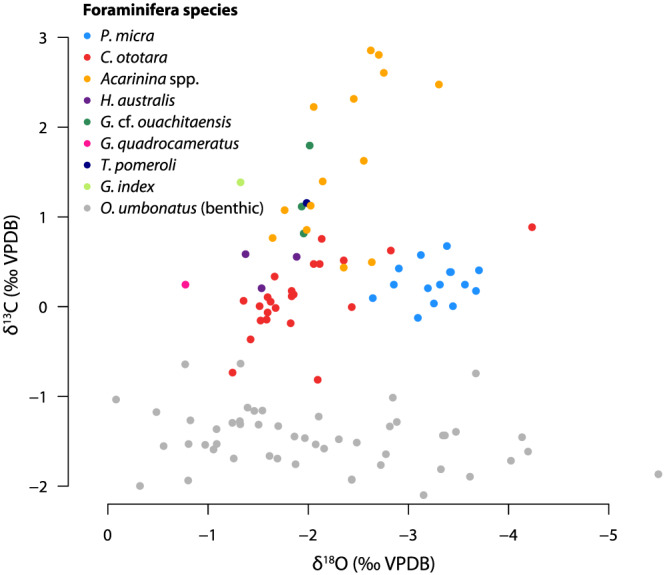
Crossplot of measured planktic and benthic foraminiferal δ^13^C (‰ VPDB) versus δ^18^O (‰ VPDB) for Site 647. Plotted data is from the interval 500–390 mbsf. Species labeling as in Figure 7.

Foraminiferal shells appear pristine under both LM and SEM (Figures 2 and 3). Furthermore, relative “isotope ordering” between the different planktonic species, as evident from cross plotting of δ^13^C and δ^18^O (Figure 8), is similar to that recorded globally for the Eocene (Pearson et al., 2001; Sexton et al., 2006). This supports the assumption that, although δ^18^O values are very low, they do reflect sea surface conditions and are not biased by diagenesis. The isotope ordering characterizes *P. micra* as a mixed‐layer nonsymbiont‐bearing calcifier with a δ^18^O signature similar to the mixed layer‐dwelling genus *Acarinina* (Sexton et al., 2006), but with a large vital effect leading to lower δ^13^C values, consistent with time‐equivalent records at tropical latitudes (Pearson et al., 2001; Wade & Pearson, 2008). The offset in carbon isotopes is likely related to the small shell size (large surface to volume ratio), and potentially rapid growth, a phenomenon seen in small surface‐dwelling species in the Holocene (Birch et al., 2013). The δ^13^C values of *Acarinina* spp. are higher, likely associated with a positive δ^13^C vital effect reflecting algal photosymbiosis (Pearson et al., 2006), and are highly variable between 0.5‰ and 3‰. The nonsymbiont‐bearing genus *Chiloguembelina* might also have been surface calcifiers (Pearson et al., 2007), but at this site, *C. ototara* records somewhat higher δ^18^O values, similar to *H. australis*. This is consistent with Eocene stable isotope values of *C. ototara* in the northwest Atlantic (Sexton et al., 2006), indicating that the species *C. ototara* might have been deeper dwelling than other species within this genus.

### 3.6 Benthic Foraminifera: Stable Isotope Chemistry

Both δ^18^O and δ^13^C of the benthic species *O. umbonatus* are very low (Figure 7). Carbon isotope ratios are between −0.5‰ and −2‰. Very high variability (between 0‰ and −5‰) is recorded in δ^18^O values, much more than in carbon isotope ratios or in planktonic δ^18^O (Figure 7). Benthic foraminifera shells, similar to the planktonics, appeared pristine under both LM and SEM (Figures 3i, 3m, and 3n). Therefore, this is unlikely to be caused by diagenesis. Our benthic foraminifera isotope records do not show clear trends, although two increases in δ^13^C are recorded around ~490–485 and ~455–448 mbsf (Figure 7).

### 3.7 Planktonic Foraminifera: Trace Elements

In the samples that yielded enough material for planktonic foraminiferal shell trace element analysis, we observed high values of contaminants, particularly Fe and Mn (Figure S1). High values of Fe and Mn are associated with authigenic carbonate concretions such as siderite (FeCO_3_), rhodochrosite (MnCO_3_), and intermediates between these at Site 647 (Arthur, Srivastava, et al., 1989). Although these concretions do not necessarily contain aberrant Mg or Ca, these results indicate that the foraminifera shell cleaning procedure likely did not remove all surface contaminants, which could include adhering clay minerals and small amounts of authigenic carbonates. We therefore consider the measured Mg/Ca values unsuitable for deriving temperature at this site but do provide them in Data Set S1. Mg/Ca values for *Acarinina* spp. and the other measured planktonic species fall within a range of 2–4 mM/M, with no clear trends through time.

## 4 Discussion

### 4.1 Depositional Setting and Sediment Sourcing

In order to establish where our recorded palynological and (organic) geochemical signals derive from, we establish the depositional setting and source of sedimentary components in the middle Eocene at Site 647. GDGT indicator ratios show that isoGDGTs were likely produced in the sea surface. brGDGTs do not seem to have been produced in the marine realm, as brGDGTs containing cyclopentane moieties are absent (Peterse et al., 2009; Sinninghe Damsté, 2016; Weijers et al., 2014). This lack of cyclic brGDGTs might indicate acidic soils in the hinterland, similar to modern tropical soils (Weijers et al., 2007). This is consistent with the inferred dominance of warm and wet mixed conifer‐broadleaf forests in the high Arctic (Eberle & Greenwood, 2012; Jahren, 2007) and on Greenland (Eldrett et al., 2009) during the middle Eocene. High values of IR_hexa_ might indicate an additional contribution by river‐produced brGDGTs (De Jonge, Stadnitskaia, et al., 2014), as soils typically show a positive correlation between the amount of cyclic brGDGTs and 6‐methyl brGDGTs (Sinninghe Damsté, 2016), which is the opposite of what we observe here. The likely terrestrial source of brGDGTs supports the decision to exclude all samples with BIT > 0.4 from TEX_86_ analysis due to a potential terrestrial overprint from soils or rivers.

Dinocyst assemblages consist of a mixture of inner shelf to oceanic taxa, of which the more proximal taxa, such as the inner shelf Goniodimidae, have likely been transported offshore. Consistent presence of the oceanic dinocysts *Impagidinium* spp. and *N. labyrinthus* likely indicates in situ production in the overlying surface waters (Dale & Dale, 1992). A transition from a more inshore assemblage to a more offshore assemblage occurs over the studied interval, with a clear increase in oceanic taxa around 455 mbsf (Figure 5). Although the palynological assemblages are predominantly marine, the abundance of (bisaccate) pollen is much higher than in regional Pliocene to recent assemblages in the Labrador Sea, including those at Site 647 (McCarthy & Mudie, 1998). In general, saccate pollen are wind transported and thus relatively more abundant in distal sediments compared to nonsaccate pollen (McCarthy & Mudie, 1998). In the modern Labrador Sea, bisaccate pollen are predominantly transported by the midlatitude westerlies and thus derive from North American land masses to the west (Mudie & McCarthy, 1994). As Site 647 was located at a similar latitude in the middle Eocene, and the north American continent might have been closer, this sourcing was likely similar in the middle Eocene.

The hinterland that supplied terrigenous clays and biomarkers to the sediment could be a combination of southern Greenland and land masses to the west, through weathering and soil erosion. Wind‐transported pollen are more likely to have dominantly come from the west, being transported by the westerlies. Overall, there are no indications for turbidites or other mass transport from the shelf in these hemipelagic sediments (Srivastava et al., 1987), which indicates it is unlikely our proxy records are disturbed by transported shelf sediments. Following the above, planktonic foraminifera and dinoflagellate cysts are interpreted to derive mainly from overlying surface waters or transported from more inshore surface waters. Benthic foraminifera are interpreted to represent in situ bottom water conditions.

### 4.2 Warm, Low‐Salinity, and Nutrient‐Rich Surface Waters in the Middle Eocene Labrador Sea

**Figure 9:**
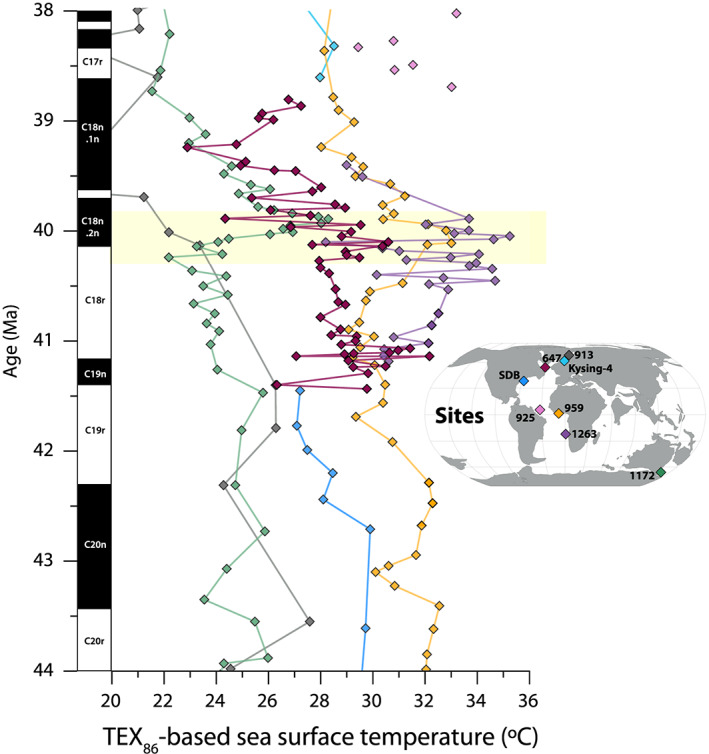
Compilation of middle Eocene TEX_86_‐based sea surface temperatures. Site 647 (red) data plotted together with published TEX_86_ data from the Atlantic basin: ODP Site 913, Norwegian‐Greenland Sea (gray) (Inglis et al., 2015, 2020; Liu et al., 2009); Kysing‐4 borehole, North Sea Basin (light blue) (Śliwińska et al., 2019); ODP Site 925, equatorial Atlantic Ocean (pink) (Liu et al., 2009); ODP Site 959, equatorial Atlantic Ocean (orange) (Cramwinckel et al., 2018); Site 1263, subtropical South Atlantic Ocean (purple) (Boscolo‐Galazzo et al., 2014); and South Dover Bridge, Atlantic coastal plain (blue) (Inglis et al., 2015). TEX_86_ record from Site 1172 (green) (Bijl et al., 2009, 2010) added as a high southern latitude end‐member. We are exclusively plotting temperatures derived using TEX_86_ ( ${\mathrm{TEX}}_{86}^{H}$ calibration of Kim et al., 2010) for optimal comparability. Propagated analytical plus ${\mathrm{TEX}}_{86}^{H}$ calibration uncertainty is ±2.6°C. Age follows GTS2012.

Middle Eocene TEX_86_‐based SSTs from the Labrador Sea are higher than 24°C throughout this record. We record two maxima with SST exceeding 30°C around ~482 and ~452 m (Figure 6a). For comparison, modern annual average SSTs in this region were about 4–8°C for the period 1982–2010 (Singh et al., 2013). Our reconstructed temperatures are comparable to alkenone‐based SSTs from the Newfoundland margin (Liu et al., 2018). We compare our TEX_86_‐based SSTs to the same proxy at a range of different localities (Figure 9). For this purpose, we plot our data on an age scale using the age‐depth constraints of Firth et al. (2012). Pre‐MECO SSTs at Site 647 are similar to those from the equatorial Atlantic Ocean and a few degrees lower than those from the (sub)tropical South Atlantic (Boscolo‐Galazzo et al., 2014; Cramwinckel et al., 2018) (Figure 9). They are distinctly higher than those from the Southwest Pacific Ocean (Bijl et al., 2010) and Norwegian‐Greenland Sea (Inglis et al., 2015, 2020; Liu et al., 2009). SSTs from the early‐middle (~48 Ma) and middle (~46 Ma) Eocene Arctic Ocean (~8–14°C) (Brinkhuis et al., 2006; Sangiorgi et al., 2008) are also much lower than the middle Eocene Labrador Sea, although coeval estimates are not available. After strong post‐MECO cooling (see section 4.5), a late Eocene warming trend (39.2–38.7 Ma) initiates in the Labrador Sea, converging to SSTs comparable to those from the nearby North Sea Basin (Śliwińska et al., 2019). In the Norwegian‐Greenland Sea, similar late Eocene warming initiates somewhat later, around 38 Ma.

Notably, TEX_86_ might be biased toward temperatures of the main season of primary productivity when export of molecular tracers to the seafloor through fecal pelleting is highest (e.g., Sluijs et al., 2006; Wuchter et al., 2006). However, in the modern North Atlantic, this is the spring (and secondarily autumn) bloom, not the summer season (e.g., Yoder et al., 1993). Moreover, there is no clear influence of seasonality on the TEX_86_‐temperature relationship in the modern core‐top data set (Tierney & Tingley, 2014), and modern water column measurements imply that the seasonal cycle in GDGT production is homogenized at depth (Richey & Tierney, 2016; Wuchter et al., 2006; Yamamoto et al., 2012). Taken together, we find it unlikely that these high Labrador Sea SSTs are biased toward the summer season.

The relatively low species diversity and dominance of thermocline‐ and subthermocline‐dwelling planktonic taxa suggests somewhat unstable surface oceanic conditions, or conditions unfavorable to mixed‐layer living planktonic foraminifera. Deeper levels of the water column, close to the base of or beneath the thermocline, were apparently more stable and/or favorable. This contrasts strongly with Holocene sediments at the location of Site 647, which contain considerably richer and more diverse planktonic foraminifera assemblages compared to the middle Eocene, despite cooler mean annual temperatures (Srivastava et al., 1987).

This could be a consequence of several factors, including closer proximity to land and higher terrigenous inputs during the Eocene, high nutrients together with high algal concentrations, reduced salinity, or high turbidity, all conditions for which planktonic foraminifera are not specialized (Bijma et al., 1990; Morey et al., 2005; Schmidt et al., 2004). The consistent presence of obligate heterotrophic protoperidinioids (Sluijs et al., 2005) at around 5% of the total dinocyst assemblage indicates that conditions were mesotrophic and food limitation likely did not play a role. The abundance of *G. eovariabilis* and *G. quadrocameratus*, which are considered to be exclusively deep dwellers (Coxall & Spezzaferri, 2018), argues for a stable subsurface water mass with a reliable food supply. These taxa were likely ecologically similar to the modern relative *Globorotaloides hexagonus*, whose abundance maxima have been shown to correlate with the nutrient‐rich subsurface water masses, both beneath the California Current (Ortiz et al., 1996) and in the equatorial Pacific (e.g., Rippert et al., 2016, 2017). The high abundance of agglutinated benthic foraminifera supports the idea of a relatively high‐nutrient flux from the surface to the deep ocean, with low oxygen content of bottom waters (Kaminski et al., 1989).

**Figure 10:**
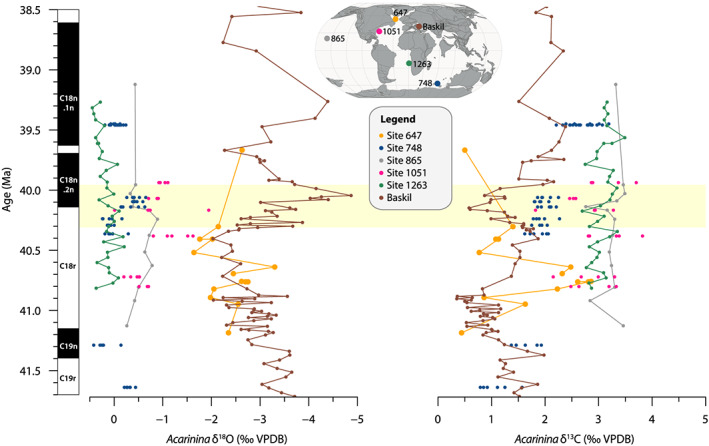
Compilation of middle Eocene *Acarinina* spp. stable oxygen (left) and carbon (right) isotope records. Site 647 (orange) data together with published data from the North Atlantic Ocean (Site 1051; pink; Edgar et al., 2013), South Atlantic Ocean (Site 1263; green; Boscolo‐Galazzo et al., 2014), Indian Ocean (Site 748; blue; Edgar et al., 2013), Pacific Ocean (Site 865; gray; Henehan et al., 2020), and Tethys Ocean (Baskil section; brown; Giorgioni et al., 2019). Data from Sites 748 and 1051 represent a size range of *Acarinina* spp. Age models for all sites have been converted to GTS2012.

Comparison of *Acarinina* spp. stable isotope ratios at Site 647 to coeval records from sites in the Atlantic and Indian Ocean establishes their relatively low values in both δ^18^O and δ^13^C (Figure 10) and reveals they are more similar to values from the Baskil section in the Tethys Ocean. Furthermore, the large offset (~1.5‰) between δ^18^O of surface dwellers *P. micra* and *C. ototara* implies strong density stratification in the upper water column. The range of planktonic foraminifera species, including taxa previously identified as surface mixed‐layer, thermocline, and subthermocline specialists, is in agreement with a (seasonally) stratified water column. Taken together with the dinocyst assemblages, which contain a very high proportion of the extinct Eocene genus *Phthanoperidinium* spp. (Figure 6c) that was adapted to lower than normal marine salinities, this suggests low‐salinity surface waters underlain by a strong pycnocline. We do note that the abundance of *Phthanoperidinium* spp. is not continuously high throughout our record, and is especially much lower in the youngest part, above the interval where we measured foraminiferal isotopes, suggesting changing conditions.

As we have reason to suspect low‐salinity surface waters, we estimate the δ^18^O of surface waters (δ^18^O_sw_) using two established δ^18^O‐temperature relationships (Erez & Luz, 1983; Kim & O'Neil, 1997). We input our TEX_86_‐based SST values in combination with planktonic foraminiferal δ^18^O_calcite_ to derive δ^18^O_sw_ (Figure 6b). As TEX_86_‐based SST is more likely to be overestimated than underestimated, the calculated δ^18^O_sw_ values based on *P. micra* provide a maximum estimate for pre‐ and post‐MECO δ^18^O of the surface waters of −1.5‰ to −0.5‰ (Figure 6b). Reduced surface salinity might have also caused the rather impoverished planktonic foraminifera assemblages, through variable or suboptimal conditions and short growth periods before reaching gametogenesis (Bijma et al., 1990).

Low SSS in the middle Eocene Labrador Sea could have been caused by intensified hydrological cycling at these latitudes under higher global temperatures, as indicated by theory and simulations under Eocene boundary conditions (Held & Soden, 2006; Pierrehumbert, 2002; Speelman et al., 2010). Additionally, influence of exported low‐salinity surface waters from further north in the sub‐Arctic and/or Arctic may have played a role. Proxy reconstructions support that the early‐middle Eocene Arctic Ocean had extremely fresh surface waters and strong salinity stratification (Brinkhuis et al., 2006; Gleason et al., 2009; Waddell & Moore, 2008). High abundances of the peridinioid dinocyst *Phthanoperidinium* spp. as recorded in our Labrador Sea record (Firth et al., 2012; this study) also occur in coeval sediments from the Norwegian‐Greenland Sea (Eldrett et al., 2004; Eldrett & Harding, 2009). Eocene palynological assemblages from the Arctic Ocean are likewise characterized by high abundance of *Phthanoperidinium* spp. and the ecologically similar genus *Senegalinium* spp. (Sangiorgi et al., 2008). Both of these genera have been inferred to be tolerant of low salinities (Barke et al., 2011; Frieling & Sluijs, 2018; Sluijs & Brinkhuis, 2009), and their high abundances at high northern latitudes in the Eocene have been linked to presence of relatively fresh surface waters (Barke et al., 2011; Sangiorgi et al., 2008) and high productivity (Eldrett & Harding, 2009). In the middle Eocene Arctic, abundance of *Phthanoperidinium* and *Senegalinium* covaries cyclically with pulses of the freshwater fern *Azolla*, underscoring the link to low salinity (Barke et al., 2011; Brinkhuis et al., 2006). Arctic surface waters might have been connected to the North Atlantic through the Nordic Seas (Brinkhuis et al., 2006; Stärz et al., 2017) or the Nares Strait, as the Bering Strait connecting to the Pacific likely had not opened yet (Hegewald & Jokat, 2013; O'Regan et al., 2011). Especially, model simulations with an open Arctic‐Atlantic connection show low SSS in northern North Atlantic, including the Labrador Sea (Hutchinson et al., 2019; Roberts et al., 2009; Tindall et al., 2010).

### 4.3 Restricted and Highly Variable Bottom Waters in the Middle Eocene Labrador Sea

**Figure 11:**
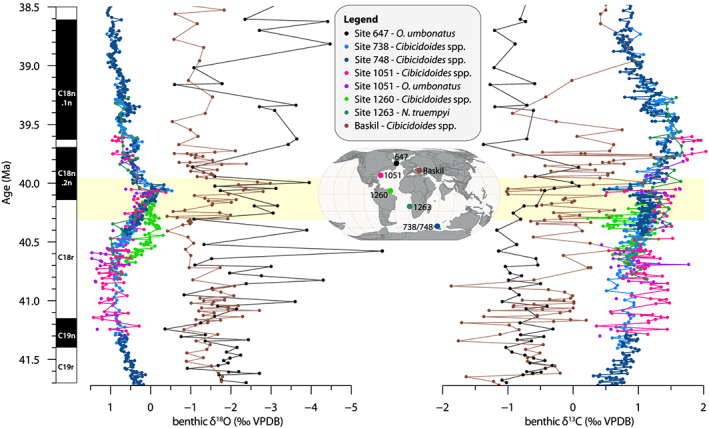
Compilation of middle Eocene benthic foraminiferal stable oxygen (left) and carbon (right) isotope records. Site 647 (black) data together with published data from the Southern Ocean (Sites 738 and 748; light and dark blue; Bohaty & Zachos, 2003; Bohaty et al., 2009), Atlantic Ocean (Site 1051 pink/purple and Site 1260 light green, Edgar et al., 2010; Site 1263 dark green, Boscolo‐Galazzo et al., 2014), and Tethys Ocean (Baskil section; brown; Giorgioni et al., 2019). Isotope values for *Oridorsalis umbonatus* (Sites 647 and 1051) and *Nuttallides truempyi* (Site 1263) have been converted to *Cibicidoides*‐equivalent values following the isotope correction factors from Katz et al. (2003). Age models for all sites have been converted to GTS2012.

We compare our benthic foraminiferal δ^13^C and δ^18^O data from the Labrador Sea to a compilation of data from previously studied sites from the middle Eocene Atlantic and Southern Ocean (Figure 11). This reveals the isotopic signature of bottom waters at Site 647 to be very different from the global deep ocean, with δ^18^O about 2–4‰ lower and δ^13^C 1–2‰ lower. The benthic records from the Labrador Sea, especially δ^18^O, are also characterized by much higher variability and are comparable only to data from the Baskil section in the Neo‐Tethys basin, although foraminiferal preservation is more varying in that record (Giorgioni et al., 2019). The middle Eocene Baskil section represents a restricted hemipelagic setting. The negative offset in both oxygen and carbon isotopes compared to the global compilation is similar to that for the late Eocene at the same site (Coxall et al., 2018). These middle‐late Eocene values are in sharp contrast to the modern north Atlantic, where deep waters are characterized instead by a “young”, high δ^13^C signature relative to the global ocean, caused by sinking of nutrient‐poor surface waters (Kroopnick, 1985).

The observed offset indicates that the middle Eocene Labrador Sea was likely not well connected to the global deep ocean. Low δ^13^C values of *O. umbonatus* point to a relatively large input of organic‐derived dissolved inorganic carbon to Labrador Sea bottom waters. This could relate either to the presence of relatively “old” waters at the end of the circulation loop (comparable to the modern North Pacific) or to local trapping of organic carbon. Given the extremely deviant Labrador Sea isotopic values, the latter option seems more likely. The TOC contents averaging 0.2% in these hemipelagic sediments indicate that not all organic matter was remineralized during sinking through the ~2000–3,000 m deep water column, further supporting poor ventilation. The above is furthermore consistent with assessments of the benthic foraminifera assemblages (Kaminski et al., 1989). Calcareous species such as *Cibicidoides* and *Nuttallides* are rare during the middle and late Eocene at Site 647, and the assemblages are dominated (80% of benthic assemblage) by agglutinated species of the “flysch‐type” (Gradstein & Berggren, 1981; Kaminski et al., 1989), that is, assemblages classically associated with Alpine foreland basins receiving high sediment and food supply and reduced bottom water oxygenation. This has previously been interpreted to signal basin restriction, limited seafloor carbonate availability, and high food supply (Kaminski et al., 1989). Regarding the highly variable and offset δ^18^O values, previous work has suggested a diagenetic overprint due to burial depth, as well as the presence of authigenic carbonates (Arthur, Srivastava, et al., 1989). However, we consider a diagenetic overprint unlikely based on our SEM observations, as these indicate a high quality of calcite preservation lacking visible signals of extensive recrystallization. Furthermore, burial diagenesis would have affected the planktonic foraminiferal δ^18^O signal similarly. Therefore, we suspect that the large variability in benthic δ^18^O represents a primary signal that records highly variable benthic conditions.

### 4.4 Implications for Regional and Global Circulation Patterns

**Figure 12:**
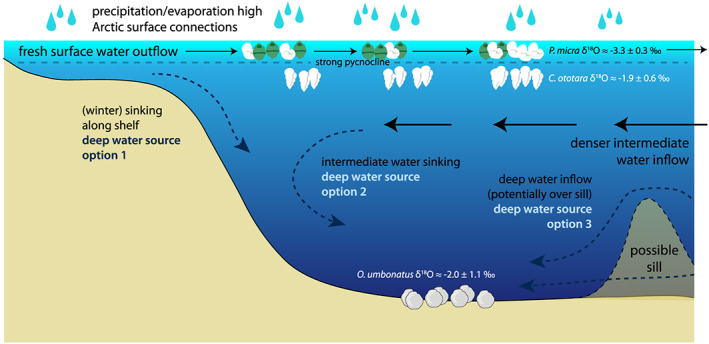
Schematic of hypothesized middle Eocene Labrador Sea circulation. Fresh surface waters are sustained by high precipitation over evaporation and/or surface water connections to the fresh Arctic. These surface waters flow out, likely to the south/east. Denser intermediate and deep waters flow in, likely from the south/east, potentially restricted by sills related to Labrador Sea spreading. Three sources of deep water are hypothesized: winter sinking along the shelf (1), sinking of inflowing intermediate water (2), and/or inflow of deep water (3).

Together, these surface and deep water conditions lead us to characterize the middle Eocene Labrador Sea as a basin with an isolated, estuarine‐type circulation. A warm, low‐salinity surface water plume lay on top of a denser, sluggishly circulating deeper water mass, which was poorly connected to the greater Atlantic Ocean (Figure 12). Such a fresh surface layer could have been sustained by a combination of factors. These factors include excess precipitation, directly and indirectly through river runoff from the surrounding land masses. Additional freshening could have occurred through surface water transport from the Arctic Ocean through shallow open Arctic‐Atlantic gateways. In the middle Eocene southern Labrador Sea, inflow of intermediate waters would be expected to compensate for shallow outflow. In terms of deep waters, if the large variability in benthic δ^18^O indeed represents a primary signal, this suggests relatively rapid changes in sourcing of deep water masses, perhaps even seasonally. One source of deep water could have been local cooling and sinking of denser plumes that cascaded down the continental slopes during winter. Another would be inflow of (southern‐sourced) deep salty water, or sinking of inflowing intermediate waters in the narrow Labrador Sea. The Reykjanes Ridge likely formed a barrier between the Labrador Sea and northeastern Atlantic (Arthur, Dean, et al., 1989). Furthermore, possible bathymetric highs associated with active east‐west spreading in the Labrador Sea might have functioned as sills, restricting deep water circulation. Although highly detailed tectonic reconstructions are not available, a basement ridge named the West Thulean Rise might have played such a role (Egloff & Johnson, 1975). The Thulean Rise feature formed around 60 Ma at the junction of mid‐Atlantic and Labrador Sea spreading, and its western half, the West Thulean Rise, subsequently moved westward with Labrador Sea spreading (Egloff & Johnson, 1975).

Overall, the strongly stratified and restricted setting we reconstruct denies the possibility of significant, globally contributing deep water forming in, or circulating into, the middle Eocene southern Labrador Sea. This implies that either no NCW formed in the North Atlantic until the late Eocene (Abelson & Erez, 2017; Coxall et al., 2018; Davies et al., 2001) or NCW formed in another source region in the North Atlantic and subsequently bypassed the southern Labrador Sea. If NCW formed in the Nordic Seas, and entered the Atlantic through GSR overflow, it may have taken a more easterly path, instead of circulating to the west through the Labrador Sea (Uenzelmann‐Neben & Gruetzner, 2018). Tectonic evolution of the North Atlantic Ocean was likely instrumental in determining when the Labrador Sea became a source region for NCW formation (Coxall et al., 2018).

### 4.5 The Signature of the MECO in the Labrador Sea

Especially given expected high‐latitude amplification of global temperature changes (e.g., Lunt et al., 2012), the recorded TEX_86_‐based sea surface MECO warming of 2°C is very subdued relative to that at low latitudes and in the Southern Hemisphere based on the same proxy (Bijl et al., 2010; Boscolo‐Galazzo et al., 2014; Cramwinckel et al., 2018) (Figure 9). Furthermore, while during the recovery of the MECO, SSTs return to preevent values at the other studied locations, the Site 647 record indicates much stronger and more prolonged cooling over the interval 40–39.3 Ma, converging to SSTs similar to those from the Southwest Pacific Ocean. We note that this compilation of SSTs suggests asynchronous warming during MECO, but this might (partially) reflect age model discrepancies on the sub‐Myr scale. Additionally, there is an incursion of the cosmopolitan dinocyst genus *Cleistosphaeridium* during the peak of the MECO (Figure 6), although we note that high abundance of *Cleistosphaeridium* spp. is not unique to only the MECO interval at Site 647. Similar poleward range expansion of plankton species from lower latitudes has been recorded in southern Indian Ocean calcareous nannofossils (Villa et al., 2008) and in Southwest Pacific dinocyst assemblages (Bijl et al., 2010; Cramwinckel et al., 2020) during the MECO.

The MECO at Site 647 is furthermore associated with signs of dissolution in planktonic foraminifera, with peak SST coinciding with full absence of all planktonic foraminifera species (dark gray horizontal band in Figure 6). As calcareous benthic foraminifera are present throughout the MECO at Site 647, and sediments retain CaCO_3_ (Arthur, Srivastava, et al., 1989), this was not related to full carbonate dissolution at the seafloor due to a rise in carbonate compensation depth—as recorded for other deep ocean localities (Bohaty et al., 2009; Sluijs et al., 2013). Instead, since the planktonic foraminifera assemblage consists mostly of small species with delicate shells, their disappearance might indicate selective dissolution at the seafloor (Berger, 1970). This could have been driven by lysocline shoaling and modest bottom water acidification in the Labrador Sea, or dissolution above the lysocline associated with high organic matter input. Alternatively or additionally, the disappearance of mixed‐layer planktonic foraminifera could have been caused by environmental exclusion, as fresh surface waters warmed and modestly acidified (Henehan et al., 2020). The latter effect would have been amplified by the higher solubility of CO_2_ in waters of lower salinity. These combined environmental changes could have resulted in adverse conditions for surface‐dwelling foraminifera.

### 4.6 A Pre‐MECO Warming Associated With Low‐Latitude Plankton Incursions

Surprisingly, peak TEX_86_ values in our Labrador Sea record were not reached during the MECO, but during a transient warming interval of 2–3°C before the MECO, around 41.1 Ma (Figure 6), which has not been recorded at other localities. This pre‐MECO warming coincides with an incursion of the unusual planktonic foraminifera *H. australis* (purple horizontal band in Figure 6) (*Hantkenina alabamensis* of Srivastava et al., 1987). The genus *Hantkenina* has been ascribed a low‐latitude affinity (Boersma et al., 1987). According to our few new data points, *H. australis* appears to be a lower mixed‐layer dweller, having a δ^18^O that is consistently higher than our surface tracers *P. micra* and *Acarinina* spp. This is consistent with previous perspectives on hantkeninid depth ecology for the middle Eocene (Coxall et al., 2000). It has been suggested that *H. australis* was somewhat more cold tolerant, having been described from the Hampden formation in southern New Zealand (Coxall & Pearson, 2006; Morgans, 2009), although multiproxy reconstructions indicate that this region was very warm at this time (SSTs > 20°C) (Burgess et al., 2008; Hollis et al., 2012).

Biotic change during the pre‐MECO warming is also evident in the dinocyst assemblages. Simultaneous with *H. australis*, an incursion of the midlatitude/low‐latitude dinocyst species *Cordophaeridium gracile* occurs (Figure 6). These dinocysts are large enough to occur in foraminifer preparations and are present in such high abundances that they physically stick to foraminifera in the sieved >63 μm fraction (Figures 3g and 3h). The genus *Cordosphaeridium* is an open marine taxon that has an affinity for high temperatures (e.g., Frieling & Sluijs, 2018). Together, these records provide strong evidence for poleward plankton migration associated with this transient warming. While higher SSTs are reached during the 41.1 Ma warming than during the MECO, planktonic foraminifera do not seem affected by dissolution. Possibly, this pre‐MECO warming represents a redistribution of heat regionally rather than a global, greenhouse gas‐driven event. For example, northward extension of the (proto‐) North Atlantic Current or shifting of the subpolar gyre with respect to Site 647 could have caused regional warming. Closer assessment of multiple sites across this time interval is necessary to reveal the spatial extent and cause of this newly recognized event.

## 5 Conclusions

Based on integration of the multiproxy reconstructions from ODP Site 647, we conclude that the middle Eocene Labrador Sea was a strongly salinity‐stratified basin, with a restricted estuarine circulation pattern. Comparable dinocyst assemblages in the Labrador Sea and Nordic Seas indicate a degree of surface connectivity over the GSR. Superimposed on these background conditions, the MECO stands out as 2°C of warming, which is muted compared to other regions, and is followed by strong cooling. Furthermore, we record another, previously undescribed and thus probably regional pre‐MECO warming of ~2–3°C around 41.1 Ma. This warming was associated with low‐latitude plankton incursions, likely representing regional oceanographic changes. The reconstructed setting, both during background climate and superimposed transients, likely precluded formation of deep waters in sufficient volume to be exported, although local winter sinking may still have occurred. This implies either no NCW formed in the North Atlantic in the middle Eocene, or alternatively NCW formed in a different source region, bypassing the southern Labrador Sea in its journey south. These results provide new constraints for models simulating middle Eocene oceanography.

## Supporting information

Supporting Information S1

Table S1

Data Set S1

## Data Availability

The data set presented here is available online (http://doi.org/10.17605/OSF.IO/Z6MAU).
