# Supplementary material for: A Warm, Stratified, and Restricted Labrador Sea Across the Middle Eocene and Its Climatic Optimum
Source: Paleoceanogr Paleoclimatol. 2020 Oct 9;35(10):e2020PA003932. doi: 10.1029/2020PA003932 (PMC7590098; doi:10.1029/2020PA003932)
Supplement: Supplementary file 1 — Supporting Information S1 [file PALO-35-na-s001.docx]

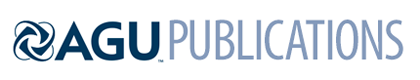


*Paleoceanography and Paleoclimatology*

Supporting Information for

**A warm, stratified, and restricted Labrador Sea across the middle Eocene and its Climatic Optimum**

Margot J. Cramwinckel^1^, Helen K. Coxall^2^, Kasia K. Śliwińska^3^, Marcel Polling^1*^, Dustin T. Harper^4#^, Peter K. Bijl^1^, Henk Brinkhuis^1,5^, James S. Eldrett^6^, Alexander J. P. Houben^7^, Francien Peterse^1^, Stefan Schouten^1,5^, Gert-Jan Reichart^1,5^, James C. Zachos^3^, and Appy Sluijs^1^

^1^Department of Earth Sciences, Faculty of Geoscience, Utrecht University, Utrecht, The Netherlands
^2^Department of Geological Sciences, Stockholm University, Stockholm, Sweden
^3^Geological Survey of Denmark and Greenland, GEUS, Copenhagen, Denmark
^4^Department of Earth and Planetary Sciences, University of California–Santa Cruz, Santa Cruz, California, USA
^5^NIOZ Royal Netherlands Institute for Sea Research, Department of Marine Microbiology and Biogeochemistry, and Utrecht University, Den Burg, The Netherlands
^6^Shell International Exploration & Production B.V, Rijswijk, The Netherlands
^7^Applied Geosciences Team, Netherlands Organisation for Applied Scientific Research (TNO), Utrecht, the Netherlands
^#^Now at Department of Geology, The University of Kansas, Lawrence, Kansas, USA
*Now at Naturalis Biodiversity Center, Leiden, The Netherlands]

**Contents of this file**

Figures S1 to S2

**Additional Supporting Information (Files uploaded separately)**

Caption for Table S1

**
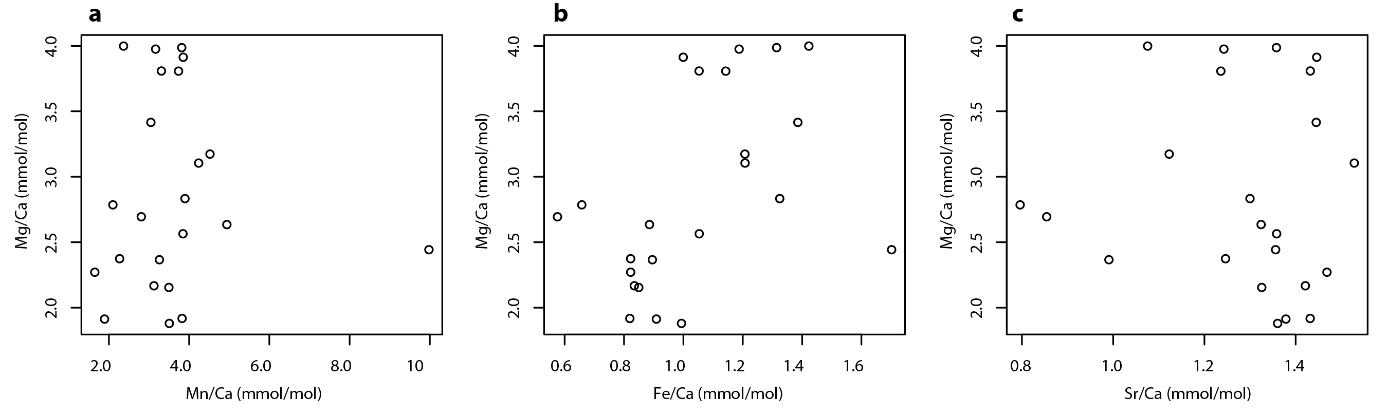
**

Figure S1. Relationships between Mg/Ca and several trace element ratios in planktic foraminiferal shells (different species ) from Site 647. a, Mg/Ca versus Mn/Ca. b, Mg/Ca versus Fe/Ca. c, Mg/Ca versus Sr/Ca. All ratios in mmol/mol.


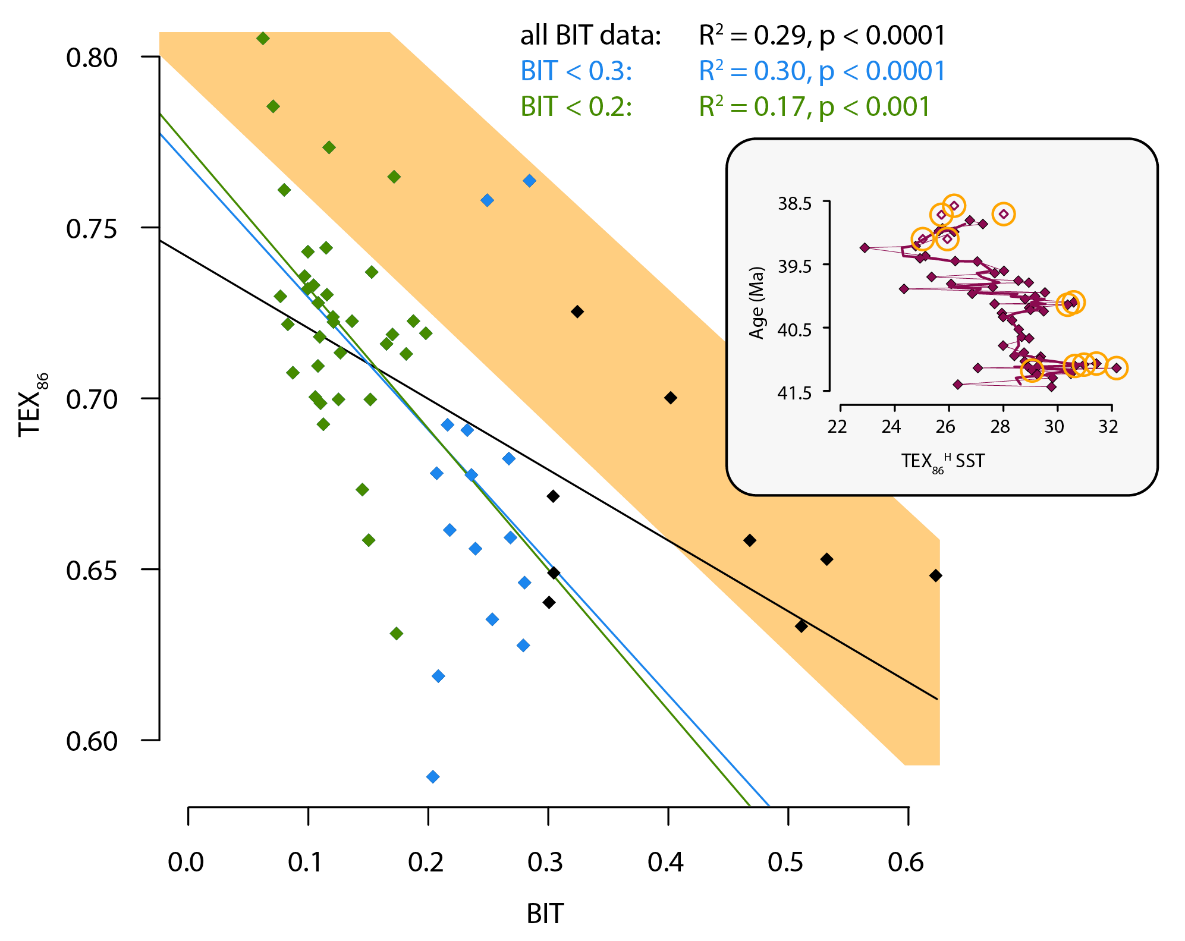


Figure S2. Relationship between TEX_86_ and BIT at Site 647, showing a significant relationship for the full dataset (p < 0.0001), but also for subsets of the data with BIT < 0.3 (p < 0.0001) and BIT < 0.2 (p < 0.001). Although cross-plotting suggests the presence of two distinct populations, the smaller population highlighted in orange seems unrelated to either sample batch (Supplementary Data) or a specific stratigraphic level (see inset, as in Figure 6a). This subset is composed of samples with either relatively high TEX_86_ (the two temperature maxima in the record) or relatively high BIT (youngest samples of the record).

[Table uploaded as separate file.]

Table S1. Planktonic foraminifera assemblage census. Species abundance was qualitatively recorded as rare (R ), few (F), common (C ), or abundant (A). Additional qualitative estimates of foraminiferal preservation state (M= moderate, G= good, Ex = excellent), the abundance of total foraminifera and foraminiferal fragments and % benthic foraminifera are also recorded. ? indicates some uncertainty in the identification of T. pomeroli at this level, requiring further study.
