# Supplementary material for: A Warm, Stratified, and Restricted Labrador Sea Across the Middle Eocene and Its Climatic Optimum
Source: Paleoceanogr Paleoclimatol. 2020 Oct 9;35(10):e2020PA003932. doi: 10.1029/2020PA003932 (PMC7590098; doi:10.1029/2020PA003932)
Supplement: Supplementary file 2 — Table S1 [file PALO-35-na-s002.docx]

| Site | Hole | Cor | Type | Section | Top (cm) | Bot (cm) | Depth (mbsf) | PF Presn. | PF abundance | PF fragments | % benthic (estimate) | *Globorotaloides quadrocameratus (?)* | *Globorotaloides eovariabilis* | *Chiloguembelina ototara* | *Dipsidriplella danvillensis(?)* | *Catapsydrax unicavus* | *Globoturborotalita sp.* | *Globoturborotalita oauchitaensis* | *Globoturborotalita cf bassriverensis* | *Subbotina cf. gortani high spire* | *Subbotina eocaena* | *Subbotina yeguaensis* | *Subbotina tecta* | *Subbotina projecta* | *Subbotina senni* | *Subbotina linaperta* | *Subbotina patagonica (coarse wall)* | *Subbotina corpulenta* | *Subbotina jacksonensis* | *Subbotina yeguensis* | *Subbotina inaequispira* | *Globigerinatheka index* | *Turborotalia pomeroli* | *Turborotalia altispiroides* | *Turborotalia frontosa* | *Turborotalita praequinceloba in fine fraction* | *Turborotalita carocselleensis ?* | *Pseudohastigerina micra in fine fractio* | *Paragloborotalia nana* | *Paragloborotalia griffinoides?* | *Parasubbotina?* | *Acarinina cf. mcgowrani or medizzea?* | *Acarinina collactea* | *Acarinina mcgowrani s.s.* | *Acarinina bullbrrooki* | *Jenisinsina columbiana* | *Morozovella cf crassatus* | *Guembelitrioides higginsi?* | *Hantkenina australis* | *Hantkenina liebusi* |
| --- | --- | --- | --- | --- | --- | --- | --- | --- | --- | --- | --- | --- | --- | --- | --- | --- | --- | --- | --- | --- | --- | --- | --- | --- | --- | --- | --- | --- | --- | --- | --- | --- | --- | --- | --- | --- | --- | --- | --- | --- | --- | --- | --- | --- | --- | --- | --- | --- | --- | --- |
| 647 | A | 41 | R | 6 | 55 | 56 | 393.44 | G-M | C | C | 10 | C | C | A | R | A | F |  |  |  | F | F | R |  | F | C |  |  |  |  |  | R |  |  |  | F |  |  | F |  |  |  |  |  |  |  |  |  |  |  |
| 647 | A | 43 | R | 6 | 62 | 64 | 412.92 | G-M | C | C | 5 | C | C | C |  | A | F |  |  |  | C | F | F | F | F | F |  | F |  |  |  | C |  |  |  | F |  |  | F |  | R | R |  |  |  | R |  |  |  |  |
| 647 | A | 45 | R | 1 | 60 | 62 | 424.70 | G-M | C | A | 30 | F | C | C |  | C | C |  |  |  | F |  |  |  | F |  |  |  |  |  |  | C |  |  |  | R |  |  | F |  |  |  |  |  |  |  |  |  |  |  |
| 647 | A | 47 | R | 5 | 49 | 51 | 449.99 | G-M | C | A | 50 | C | C | R |  | C | C | C | F | F | C | R | R |  | C |  |  |  |  |  |  | R | ? |  |  |  | C |  | F |  |  |  |  |  |  |  |  |  |  |  |
| 647 | A | 49 | R | 1 | 55 | 57 | 463.35 | G-Ex | A | F | 10 | C | C | F |  | A |  | F | F | F | A |  |  |  |  |  |  | A |  |  |  |  | A? |  |  |  | C | C |  |  |  | F |  |  |  |  |  |  |  |  |
| 647 | A | 49 | R | 6 | 40 | 42 | 470.70 | G-M | C | F | 5 | F | C |  |  | C |  |  | ? |  | C |  |  |  |  |  |  |  |  |  | F |  | A |  |  |  |  |  | C |  | F |  |  | C | F |  |  |  |  |  |
| 647 | A | 50 | R | 2 | 76 | 78 | 474.76 | G-M | C | F | 5 |  |  | R |  | C |  | F | C |  | F |  |  |  |  |  | F | C |  |  |  |  | A |  |  |  |  |  |  |  |  |  |  |  |  |  |  |  |  |  |
| 647 | A | 50 | R | 6 | 59 | 61 | 480.59 | G-Ex | A | F | 2 | C | F | R |  | C |  |  | C | F | C |  |  |  |  |  | F | F | R | F |  |  | A |  | F | R | R | F | F |  |  | R | R |  |  |  |  |  | C | F |
| 647 | A | 51 | R | 2 | 115 | 117 | 484.75 | G-M | A | F | 2 | C | F | F |  | C |  |  | C? | F | A |  |  |  |  |  | F | C |  |  |  |  | A |  |  |  |  | F |  | F |  | F |  |  |  | C | R |  |  |  |
| 647 | A | 51 | R | 6 | 74 | 76 | 490.34 | G-M | A | F | 5 | C | F | F | R | C |  | F | F |  | C |  |  |  |  | F |  |  |  |  |  |  | A |  | F |  | R | F |  |  |  |  |  |  |  | R |  |  |  |  |
| 647 | A | 52 | R | 4 | 70 | 72 | 497.00 | G-M | A | F | 5 | C | F |  |  | F |  |  | C |  | C |  |  |  | F |  |  |  | C |  |  |  | A |  | F |  |  | R | C | F |  | F | C |  |  | R |  |  |  |  |
| 647 | A | 52 | R | 6 | 125 | 127 | 500.55 | G-M | A | F | 5 | C | C | R |  | C |  |  | F? |  |  |  |  |  |  |  |  |  |  |  |  |  | A |  | F |  |  | F | F |  |  | F | C |  |  |  |  | R |  |  |
